# Cognitive impairment among older adults living in the community and in nursing home in Indonesia: a pilot study

**DOI:** 10.1590/1980-5764-DN-2022-0012

**Published:** 2022-07-29

**Authors:** Rahmi Setiyani, Asep Iskandar

**Affiliations:** 1 Universitas Jenderal Soedirman, School of Nursing, Faculty of Health Sciences, Banyumas, Central Java, Indonesia.

**Keywords:** Cognitive Dysfunction, Frail Elderly, Nursing Homes, Disfunção Cognitiva, Idoso Fragilizado, Casas de Saúde

## Abstract

The demographic phenomenon of population aging has brought some consequences, including a higher prevalence of cognitive impairment.

**Objective:**

This study aimed to assess and compare cognitive impairment and its risk factors between older persons living in the community and in nursing home in Indonesia.

**Methods:**

A cross-sectional study was employed among 99 older adults living in the community and 49 nursing home residents. Cognitive function was assessed using the Mini-Mental State Examination (MMSE).

**Results:**

Older people living in the community showed a higher score on MMSE than those living in nursing home (p=0.044). Age, marital status, education level, and literacy status were significantly related to the cognitive function of older adults living in the community (p=0.003, p=0.007, p=0.005, p=0.001, respectively), while gender, education level, and literacy status were significantly related to that of nursing home residents (p=0.012, p=0.004, p=0.001, respectively).

**Conclusions:**

Older adults living in the nursing home were more likely to experience cognitive decline than their counterparts in the community. Factors associated with cognitive decline differ between community-dwelling older adults and nursing home residents.

## RESUMO

O fenômeno demográfico do envelhecimento da população trouxe algumas consequências, incluindo uma maior prevalência de comprometimento cognitivo.

**Objetivo:**

Este estudo teve como objetivo avaliar e comparar o comprometimento cognitivo e seus fatores de risco entre os idosos que vivem na comunidade e no lar de idosos na Indonésia.

**Métodos:**

Um estudo transversal foi empregado entre 99 idosos que vivem na comunidade e 49 residentes de casa de repouso. A função cognitiva foi avaliada usando o *Mini-Mental State Examination* (MMSE).

**Resultados:**

Os idosos que vivem na comunidade mostraram uma pontuação mais alta no MMSE do que aqueles que vivem em casa de repouso (p=0,044). Idade, estado civil, nível de educação e alfabetização estavam significativamente relacionados à função cognitiva de idosos que vivem na comunidade (p=0,003, p=0,007, p=0,005, p=0,001, respectivamente), enquanto gênero, nível educacional e alfabetização estavam significativamente relacionados aos idosos residentes da casa de repouso (p=0,012, p=0,004, p=0,001, respectivamente).

**Conclusões:**

Os idosos que vivem na casa de repouso tinham maior probabilidade de experimentar um declínio cognitivo do que seus colegas na comunidade. Os fatores associados ao declínio cognitivo diferem entre os idosos que habitam a comunidade e os residentes da casa de repouso.

## INTRODUCTION

Globally, the number of older people is increasing at a faster rate than all other age groups^1^. This rapid aging demographic transition has resulted in greater levels of cognitive decline, which is a growing public health issue. Previous studies demonstrated that about half of older people experienced a decline in cognitive function as part of the aging process^2,3^.

The range of cognitive function decline in the older population encompasses normal aging, mild cognitive impairment (MCI), and severe cognitive impairment^4^. Changes in cognitive abilities that occur as a normal part of the aging process should not impair an older person’s abilities to perform daily life activities^5^. However, the changes can progress at different rates, with many individuals suffering from cognitive decline severe enough to interfere with their ability to perform activities of daily living, the later diagnosed as dementia^5^.

Studies on cognitive function in older adults in different countries have predominantly identified sociodemographic and health characteristics as risk factors^6–8^. Among demographic variables, place of residence has not been much investigated. Living arrangement has been an emerging aging-related issue. In general, in most countries, most older people lived in private households and only small number of them lived in institutional settings^9^. However, changes in living arrangements of older people have occurred in many regions, and the number of older people living in an institution is expected to increase^9^.

The place of residence has been linked to the health status, well-being, and quality of life of older people^10–12^. Few previous studies have compared cognitive function and associated risk factors of older adults who live at home and in an institution. However, respondents of this study were those with MCI and dementia, not aging-related decline^13,14^.

Like many other countries, Indonesia is also experiencing a rapid increase in the older population^1^. Regarding living arrangement, most of the older people in Indonesia lived in a multigenerational household and only a minority lived in institutional settings^15^. Few studies have examined the cognitive functioning of older people in Indonesia^16,17^. However, there is a lack of studies comparing the cognitive function of older adults across the different living settings. Thus, this study aimed to identify and compare the cognitive decline and risk factors between older people living in the community and those living in nursing home in Indonesia.

## METHODS

### Settings and participants

This is a descriptive analytical study using a cross-sectional method. The study was conducted from June to September 2019. This study was conducted among older people who live in the community and in nursing home in Banyumas Regency, Central Java Province, Indonesia.

Based on the 2019 Indonesian Population Census, the population of older people in Banyumas accounted for about 236,193 people, or approximately 14% of its total population, a bit higher than the national average (9.6%). Most older people in Banyumas live in the community, and only a few live in nursing homes. There are two nursing homes in the regency: one is a private religious-based, and the other is a state-owned nursing home, with a total number of 147 residents in 2019.

The sample size was calculated using a simple formula for a pilot study^18^. An online calculator is available at http://www.pilotsamplesize.com. With a confidence level of 95% and a probability of 0.068 based on the previous study in Indonesia^19^, the sample size was small with 43 subjects in each group. Considering the low response rate based on our previous study (≤50%) (unpublished), a total of 100 community-dwelling older adults and 100 nursing home residents were invited to participate in the study.

The community samples were conveniently selected from the participants of Posyandu Lansia (integrated health service post for older people) in the nearby area, following the recommendation by cadres (community volunteers). A total of 100 community-dwelling older people were visited at their homes, and 99 of them were eligible for this pilot study. Meanwhile, the nursing home samples were conveniently recruited from the state-owned nursing home. The nursing home has a total of 100 residents, but only 49 of them met the study criteria. Eligible participants were no less than 60 years old, willing to participate, and able to speak the language(s) used to administer (Bahasa and/or Javanese). Participants who had visual or auditory impairment or active psychiatric symptoms that preclude them from completing the assessment were excluded from the study.

### Instruments

The studied variables were cognitive function, health status, and demographic characteristics. Cognitive function was assessed using the Mini-Mental State Examination (MMSE) that consists of the following sub-functions: orientation, registration, attention and calculation, repetition/recalled, and language^20^. MMSE score ranges from 0 to 30. The higher the score, the better the cognitive functioning. An MMSE score <24 indicates cognitive deficits.

This study used the adapted version of the MMSE in Indonesian language^21^. In this validity study, the tool was adapted and translated into several languages, including Bahasa and Javanese, using the procedure back-translation. The tool showed optimum sensitivity using a similar cutoff value of 24.

Health status characteristics included blood pressure, nutritional status, smoking, and illness history. Blood pressure measurement was conducted at the beginning of the research interview and then classified into “normal (normotension)” or “hypertension” using JNC 7 algorithm^22^. Nutritional status was determined by measuring body mass index (BMI) and then classified into normal or abnormal (underweight, overweight, obese, or extreme obese)^23^. Smoking and illness history referred to any cigarettes consumption and presence of any cardiovascular/neurological/metabolic diseases in the past 6 months as reported by the respondents.

Meanwhile, demographic variables included age, sex, marital, educational, literacy, and living arrangement status.

### Ethical consideration

Before collecting the data, respondents were given an explanation about the aim and nature of this study, and they signed an informed consent form if they agreed to participate. The five rights of human subjects in the research, including self-determination, privacy, dignity, anonymity, and confidentiality, were maintained throughout the study. This study has gained an ethical approval from the Health Research Ethics Committee no. 2516/KEPK/V/2019 dated May 29, 2019.

### Data analysis

The demographic and health status characteristics of respondents were described using frequency tables of categorical variables and descriptive statistics of numerical variables. Chi-square tests were used to compare the categorical variables. Fisher’s exact tests used as an alternative to the chi-square tests when one or more cells had expected count of less than 5 (i.e., presence of cardiovascular/neurological disease). Independent t-tests compared the means between two unrelated groups on the same continuous variables. Mann-Whitney U tests were used as an alternative to independent t-tests when variables followed the non-parametric distribution. Spearman’s rank tests were used instead of Pearson’s correlation to measure the correlation between two continuous variables, which were not normally distributed. This study used p≤0.05 to define the level of statistical significance. Data processing was carried out using the IBM SPSS version 25.0 software.

## RESULTS

Older people living in both the community and nursing homes had relatively similar characteristics in terms of gender, years of education, and literacy, but were significantly different in terms of age and marital status (Table 1). Older people in both groups were more likely to be of female gender with less years of education (<9 years) and literate. However, nursing home residents were significantly older and more likely to be single (i.e., widowed) than their community-dwelling counterparts (p=0.029 and p=0.000, respectively).

**Table 1:** Demographic and health status characteristics of respondents.

Age, mean±SD		Community-dwelling (n=99)	Institutional-dwelling (n=49)	p-value
		68.01±6.910	71.71±9.381	0.029*,+
Gender, n (%)	Male	26 (26.3)	18 (36.7)	0.190§
	Female	73 (73.7)	31 (63.3)	
Marital status, n (%)	Married	59 (59.6)	3 (6.1)	0.001§,+
	Single (unmarried, widowed)	40 (40.4)	46 (93.9)	
Years of education, years, n (%)	≥9	13 (13.1)	11 (22.4)	0.148§
	<9	86 (86.9)	38 (77.6)	
Literacy, n (%)	Literate	78 (78.8)	35 (71.4)	0.321§
	Illiterate	21 (21.2)	14 (28.6)	
Blood pressure, n (%)	Normal	39 (39.4)	19 (38.8)	0.942§
	Hypertension	60 (60.6)	30 (61.2)	
BMI, n (%)	Normal	57 (57.6)	35 (71.4)	0.102§
	Abnormal	42 (42.4)	14 (28.6)	
Smoking status, n (%)	Not smoking	84 (84.8)	41 (83.7)	0.853§
	Smoking	15 (15.2)	8 (16.3)	
Cardiovascular/neurological/metabolic disease, n (%)	No	92 (92.9)	46 (93.9)	1.000||
	Yes	7 (7.1)	3 (6.1)	

SD: standard deviation; BMI: body mass index

* Mann-Whitney U test

+ p≤0.05

§ Chi-square test

|| Fisher’s exact test.

In terms of health status, both older adults living in the community and nursing home had relatively similar characteristics (Table 1). Most of them had normal BMI, did not smoke, and did not have cardiovascular/neurological/metabolic diseases. However, about two-thirds of both groups had high blood pressure (hypertension).

The total MMSE score of nursing home residents was significantly lower than those of community-dwelling older people (p=0.044). The two groups showed a significant differences in the language function (p=0.004) (Table 2). When cognitive decline was defined as MMSE<24, it was found that it was present in 20.2% and 44.9% of older adult living in the community and in nursing homes, respectively (p=0.002).

**Table 2:** Cognitive functioning.

	Community (n=99)	Nursing home (n=49)	p-value
MMSE score (mean±SD)	24.09±5.043	22.59±5.082	0.044*,+
Orientation	8.20±1.969	8.00±2.363	0.798*
Registration	2.79±0.689	2.86±0.408	0.797*
Attention and calculation	3.23±1.640	2.71±1.696	0.083*
Recalled	2.24±1.001	2.20±0.979	0.727*
Language	7.63±1.549	6.82±1.787	0.004*,+

MMSE: Mini-Mental State Examination; SD: standard deviation

* Mann-Whitney U test

+ p≤0.05.

There were factors related to MMSE scores among older adults living in the community and in nursing home (Table 3). Results showed that education level and literacy status were significantly related to the cognitive function of older adults in both groups (p=0.005, p=0.001 and p=0.004, p=0.001, respectively). Better educated and literate older adults are more likely to have MMSE score above or very close to the threshold (≥24).

**Table 3:** Factors related to cognitive function among older people living in community and nursing home residents.

		Community (n=99)		Nursing home (n=49)	
		MMSE score (mean±SD)	p-value	MMSE score (mean±SD)	p-value
Age (correlation coefficient)		r=-0.298	0.003*,+	r=-0.270	0.061*
Gender	Male	24.69±4.038	0.804§	24.94±4.277	0.012+,||
	Female	23.88±5.364		21.23±5.071	
Marital status	Married	25.27±4.046	0.007+,§	26.33±2.887	0.185||
	Single (unmarried, widowed)	22.35±5.860		22.35±5.117	
Years of education (years)	≥9	27.08±2.397	0.005+,§	26.27±4.474	0.004+,§
	<9	23.64±5.190		21.53±4.786	
Literacy	Literate	25.55±3.410	0.001+,§	23.89±5.184	0.001+,§
	Illiterate	18.67±6.374		19.36±3.054	
Blood pressure	Normal	25.15±3.957	0.145§	21.58±5.480	0.370§
	Hypertension	23.40±5.561		23.23±4.797	
BMI	Normal	24.35±4.658	0.735§	22.77±5.292	0.700||
	Abnormal	23.74±5.561		22.14±4.672	
Smoking status	Not smoking	23.99±5.180	0.818§	22.37±5.333	0.487||
	Smoking	24.67±4.304		23.75±3.576	
Cardiovascular/neurological/metabolic disease	No	23.98±5.146	0.607§	22.46±5.124	0.471||
	Yes	25.57±3.309		24.67±4.726	
Living arrangement	Multi-generation	24.32±4.964	0.318§		
	Independent living	23.27±5.347			

BMI: body mass index

* Spearman’s rank test

+ p≤0.05

§ Mann-Whitney U test

|| Independent t-test.

Age had a significant negative correlation with MMSE scores, but only among community-dwelling older people (p=0.003, cc=-0.298). The effect of marital on cognitive functioning was also demonstrated by this group. Married older people showed higher MMSE scores above the threshold (≥24) compared to their counterparts (p=0.007). Meanwhile, a significant difference in MMSE scores between gender was only found among those living in nursing home (p=0.012). Male older people showed a higher score above the threshold than their female counterparts.

## DISCUSSION

This pilot study determined cognitive decline among older people in two different living settings, that is, community and nursing home, and their related characteristics. Findings showed that older adults living in nursing home showed a lower cognitive function than those living in the community. The results from this study support a previous study which reported that older people who admitted to nursing homes showed a greater cognitive decline after their admission than those who remained at home^24^.

The reasons for the decline are still unclear, but are probably linked to physical and psychological consequences of living in institution on older people. A previous study reported that many long-term care residents suffered from depression due to perceived inadequacy of care^25^. A previous study in Indonesia indicated that admitting older people in nursing home is unusual due to the perception that it is against the cultural values of filial obligation as well as having a detrimental effect on older people’s physical and psychological health^26^.

However, it is also highly possible that the decline was not a result of institutionalization. Older adults might have already been suffering from cognitive decline when they were admitted to the nursing home. A previous longitudinal study found that dementia was the strongest predictors of living in institution in old age^27^. A previous systematic review also suggested that cognitive impairment was one of the main underlying conditions of nursing home placement^28^. The causal relationship between cognitive decline and institutionalization in this study, however, could not be determined due to the study design.

The difference in cognitive decline between community-dwelling older adults and nursing home residents could also be explained by the difference in respondents’ characteristics, namely, age and marital status. In this study, nursing home respondents were significantly older than their counterparts in the community. Cognitive function generally declines with age among older adults^29^. However, after controlling the living setting, age was associated with cognitive decline in community-dwelling older adult, but not in nursing home residents. This finding is in accordance with a previous study conducted among nursing home residents that found no significant association between age and cognitive decline^30^.

Regarding marital status, in this study, nursing home residents were also more likely to be single than their community-dwelling older people. Previous studies demonstrated that marriage was related to a reduced likelihood of having cognitive decline^31,32^. Marriage has been suggested as having psychological benefits, which protect individuals from cognitive decline in later life. Married individuals would have more cognitive and social engagement and experience less loneliness and psychological distress^32^. It has been indicated in previous studies that high levels of distress and loneliness were related to a decline in cognitive ability among older people^33,34^. However, like the age variable, after the living setting was controlled, marital status was related to cognitive functioning only in community respondents. The lack of association between marital status and cognitive decline among nursing home residents in the present study was possibly related to the fact that married individuals who lived in nursing homes could be considered “single” without the presence of their spouse. Thus, it seems that not the relationship status but the meaningful social interaction affects the cognitive function. However, it warrants further investigation since these data were not available.

The female gender was associated with lower cognitive functioning among nursing home residents. Some studies have found gender differences in cognitive functioning. They found that gender discrepancies were suggested to involve complex interactions with other factors, for example, education period, specific cognitive domains, genetic vulnerability, and hormonal status^35–37^. In another study, hypertension and stroke accounted for gender differences in cognitive decline in women and men, respectively^29^.

Shorter periods of education and illiteracy were related to declines in cognitive functioning in both community- and institutional-dwelling older people. The protective benefits of education and literacy on cognitive performance have been demonstrated in several studies^6,7,17^. Poorer cognitive functioning among lower educated and illiterate older adults is possibly due to the fact that they are relatively lacking in cognitive stimulation. A previous study suggested that the length of education had a considerable impact on cognitive ability in relation to the individual’s work situation, socioeconomic status, and social activity^35^.

Interestingly, none of the health status indicators in the present study were associated with cognitive function. Previous studies showed contrary results, which found that high blood pressure, obesity, smoking, and chronic diseases, including diabetes, heart disease, and stroke, have all been suggested to have an influence on cognitive function^5,38–40^. Further research in this area is required.

This study has several limitations:

1. The number of respondents in both groups was not equal due to the limited number of nursing home residents who met the study criteria;
2. The cross-sectional design cannot determine a cause-effect relationship between variables; and
3. This study only investigated a few factors, although there might be other factors that affect cognitive functioning in older adults.

This study suggests that older people living in nursing home presents a more significant cognitive decline than those living in the community. Female gender, shorter years of education, and illiteracy were related to lower cognitive function among nursing home residents, while advanced age, not being married, shorter years of education, and illiteracy were related to that of community-dwelling older people. Health promotion strategies to prevent further cognitive decline should be focused on those vulnerable sub-groups.
